# Galen’s Typology of Organs

**DOI:** 10.1515/apeiron-2024-0077

**Published:** 2025-02-19

**Authors:** Dmitry Ezrokhi, Orly Lewis

**Affiliations:** Department of Classical Studies, 26742 Hebrew University of Jerusalem, Jerusalem, Israel

**Keywords:** Galen, anatomy, muscles, physiology, ancient medicine, fibres

## Abstract

This paper examines Galen’s insistence that the stomach and heart, despite their anatomical and physiological similarities to muscles, are not muscles. Through analyzing key passages in Galen’s works, we show that this claim is rooted in a consistent tripartite distinction between organs: Psychic Moving Organs (muscles), Natural Moving Organs (e.g. stomach, heart), and Natural Immobile Organs (e.g. liver, kidneys). We argue that this classification is grounded in anatomical differences between flesh and fiber that Galen deems salient enough to support further physiological explanations and corroborate his philosophical-psychological commitments. By tracing the empirical foundations and theoretical motivations for these distinctions, we shed light on the relationship between Galen’s anatomical practices and his physiology and psychology.

## 1 Introduction

Throughout his corpus, Galen consistently emphasizes his anatomical and physiological insights into the nature and function of muscles (μύες). In the treatises specifically dedicated to muscles, namely *The Anatomy of Muscles* and *The Motion of Muscles*, as well as in his comprehensive dissection manual *Anatomical Procedures*, Galen asserts his superior ability to identify, describe, and differentiate muscles.1His motivation for writing *Anatomy of Muscles* rests on his discontent with the muscular anatomy of Lykos (*Musc.Diss*. 1 [Garofalo\Debru 118,1 – 119,20 = XVIIIb 926 – 928 K.]). Galen places an impressive catalogue of his corrections and improvements on past muscular anatomies near the beginning of his *Anatomical Procedures* (*AA*. I 3 – 4 [I 13,23 – 31,17 Garofalo = II 227 – 243 K.]). We know that a similar attitude was apparent also in the earlier *Anatomical Procedures* published in two books and incorporated in the early books of *The Function of the Parts of the Human Body* (e.g., *UP*. II 7 [I 84,14 – 20 Helmreich = III 115 K.], and references in Garofalo 1994, 1796). Translations and abbreviations of the titles of Galen’s works follow Singer and Rosen 2024, 675 – 91. References to Galen include the edition consulted and the equivalent pages in Kühn 1821 – 33, abbreviated as K. His anatomical precision is further reflected in his physiological descriptions, where he employs anatomical typologies of muscular structures in his investigation of muscular activities and functions.2See for example the long discussion about the motions of antagonist muscles in *The Motion of Muscles* (*Mot.Musc*. I 9 [19 – 24 Rosa = IV 412 – 418 K.]). Following Singer and Rosen 2024, we translate the terms χρεία, δύναμις and ἐνεργεία as function, capacity and activity respectively. For arguments against this translation choice, which we cannot address here, see Havrda 2024. See also Furley and Wilkie 1984, 58 – 70.

The stomach and heart, however, consistently challenge Galen’s certainty about muscles. Throughout his writings, he acknowledges striking anatomical and physiological similarities between these organs and muscles. Yet he adamantly maintains that these two organs cannot be classified as muscles. Given this tension – his recognition of the muscle-like characteristics of the stomach and heart alongside his firm rejection of their muscular identity – his reasons for this distinction warrant closer examination.

In this paper, we explain Galen᾽s insistence on the non-muscular status of the stomach and heart. We argue that his position becomes comprehensible when viewed through a coherent typology of internal organs that he employs across various argumentative contexts, though typically without explicit introduction or justification.3We distinguish typology from classification. We understand the latter in terms of a systematic *diaresis* explicitly argued and set out by Galen, such as the distinction between uniform and non-uniform parts, the different kinds of pulse or diseases (Salas 2019; Lewis 2022). The former denotes less explicit but nonetheless operative categories in Galen’s thought – groupings through which he analyzes various anatomical, physiological, and psychological issues without fully explicating them. We therefore use the terms ‘category’, ‘group’, ‘class’ and ‘type’ interchangeably assuming a typological meaning for all of them. According to this framework, not all moving organs in the body are muscles. Rather, muscles constitute only one subclass of moving organs (what one may term Psychic Moving Organs), while the stomach and heart belong to a different group, which we term Natural Moving Organs.4While ‘natural moving organs’ does not appear verbatim in Galen, he distinguishes between muscles and ‘natural organs’ (φυσικὰ ὄργανα) when discussing ‘moving organs’ (κινούμενα ὄργανα, see T1 below). Since not all natural organs are moving organs, as we argue below, ‘natural moving organs’ designates those natural organs that are also moving organs. Given that we are working with a typology rather than a classification, the absence of explicitly named categories in Galen’s words is less significant than their utility in illuminating Galen’s thought. We thank the anonymous reviewers for pressing us on this point. Since muscles are the only organ we label as Psychic Moving Organs, we generally refer to them simply as muscles in this paper. Galen frequently contrasts these two types with a third group – Natural Immobile Organs – which includes the liver, kidneys, spleen, lung (see Table 1). We demonstrate that this tripartite division rests on a complex web of anatomical, physiological, and philosophical arguments and conceptions that both presuppose and bolster one another.

**Table 1:** Three kinds of organs – an overview.

Psychic moving organs	Natural Moving Organs	Natural Immobile Organs
Muscles	Heart Stomach Intestines Uterus Bladder	Liver Spleen Kidneys Lung

Arteries and veins are also natural moving parts by means of the same mechanism we describe below (natural capacities acting in and through fibers), but since they are not organs, but uniform parts we do not include them in the table (*Morb.Diff*. 3 [VI 841,2 - 3 K.], and with some important reservations in* Inaeq.Int*. 2 [144,13 García Novo - 145,5 = VII 735 K.]).

Scholars have noted parts of this typology, namely the distinction between muscles and Natural Moving Organs. However, the distinction has not been investigated, nor has the tripartite typology been recognized as a whole.5E.g., Meyer-Steineg 1911, 176 – 181; Debru 1996, 102 – 107 and, most recently, Salas 2024, n. 48. Therefore, we examine Galen’s typology to understand its foundations, motivations and rationale. In so doing, we shed light on and deepen our understanding of Galen’s typology of body parts, the criteria he employs, and their relationship to his broader physiological and psychological frameworks and conceptions of body and soul. This, in turn, leads us to a new appreciation of the complicated relationships between anatomical observation and physiological/psychcological speculation in Galen’s thought and argumentation.

In Section 2, we discuss the passages in which Galen confronts the anatomical evidence that might lead one to classify the heart and the stomach as muscles. In Section 3, we introduce the distinction between muscles and Natural Moving Organs, highlighting the most important commonality between them, namely fiber-induced motion. Subsequently, we examine Galen’s rich array of reasons for rejecting the identification of Natural Moving Organs with muscles: first the anatomical ones (Section 3) and then the physiological ones (Section 4). In Section 5, we turn our attention to Natural Immobile Organs and their status vis-à-vis muscles and Natural Moving Organs. In Section 6 we conclude by integrating our discussion into a broader understanding of the role of anatomy in Galen’s more theoretical elaborations.

## 2 The Stomach and the Heart

The stomach and the oesophagus form, according to Galen, one contiguous whole composed of two layers or tunics (χιτῶνες), each with a distinct fiber arrangement (ἴνες). According to Galen, the outer tunic of the stomach is invested with transverse and oblique fibers. The inner coat, on the other hand, has straight fibers.6*Nat.Fac*. III 8 (222 – 226 Helmreich = II 168 – 172 K.) For a fuller account of gastrointestinal fibers and their role in digestion, see Lewis 2023; Pelavski, Marroquín-Arroyave, Milgram and Lewis 2024. In book 10 of his *Anatomical Procedures*, when describing the outer tunic of the stomach, Galen mentions that it becomes fleshier as it proceeds caudally from the oesophagus toward the fundus of the stomach. This renders the second tunic of the oesophagus and stomach ‘closer to the nature of flesh’ (*aqrab min ṭabīʿat al-laḥm*) than the inner one and it has ‘a higher amount of blood’ (*al-khārijah al-dam fīhā akthar*).7*AA*. X 9 (78, 12 – 17 Simon). The latter part of book 9 and the whole of books 10 to 15 of *Anatomical Procedures* survive solely in Ḥubayš ibn al-Ḥasan’s Arabic translation. Throughout the paper, translations of Greek and Arabic are our own. We thank Elizur Gluck for help with the Arabic. This is already a telling remark since having flesh and being fleshy (σαρκώδης) is a defining characteristic of muscles.8Cf. *Temp*. II 5 (69,25 – 27 Helmreich = I 621 K.).

An additional, although here tacit, similarity between the oesophagus and stomach to muscles is the fact that they move. In Galen’s understanding of digestion physiology, when deglutition occurs, the stomach pulls the oesophagus along the straight fibers towards itself, shortening it, raising the larynx, and facilitating the passage of food. The oblique fibers of the outer tunic allow the stomach to contract around the food when digesting and its transverse fibers facilitate peristaltic motions expelling already elaborated food to the intestines.9*Nat.Fac*. III 8 (223,18 – 227,15 Helmreich = III.170 – 174 K.); *Mot.Dub*. 11 (167 – 177 Nutton). Cf Lewis 2023. Given these similarities, it is not surprising that Galen determines that its outer and fleshy tunic has ‘a substance [that is] is very close to the substance of muscle’ (*jawharuhā qarīb jiddan min jawhar al-ʿuḍlah*).10*AA * X 9 (78,16—17 Simon): جوهرها قريب جدا من جوهر العضلة. 

Similarly, in book 7 of *Anatomical Procedures*, Galen compares the material constitution and movements of the heart to muscles, admitting that ‘to the careless observer the substance of the muscle and the heart will seem not to differ at all.’11*AA*. VII 8 (II 431,29–3.2 Garofalo = II 610 K.): ὅστις δ’ ἀμελῶς ὁρᾷ, δόξει τούτῳ μηδὲν διαφέρειν οὐσίαν μυὸς καὶ καρδίας. Here, again, besides the apparent anatomical similarity, the additional fact that both the heart and the muscles move only strengthens the impression that they belong to the same category of body parts. However, he emphasizes that despite these similarities, the heart is not a muscle. He makes comparable observations about the similarity of the heart to a muscle in *The Distinct Types of Pulse* and *The Motion of Muscles*.12*Mot.Musc*. I 3 (5,14 – 25 Rosa = 4.377 K.); *Diff.Puls*. IV 8 (VIII 738.6 – 10 K.). He even discusses the heart and the oesophagus together as organs that tend to be viewed as muscles by other physicians and anatomists: ‘Those thinking that the heart is a muscle have committed a grave error, while those who consider the neck of the stomach to be of exactly the same substance as muscles have committed a lesser one, since its outer layer, the one with transverse fibers, is closer to the substance of muscles [sc. than the heart]. Nonetheless, even it is not strictly speaking a muscle.’13*AA*. VII 8 (II 437,19 – 25 Garofalo = II 614 K.): μέγιστα μὲν οὖν ἡμαρτήκασιν οἱ νομίζοντες μῦν εἶναι τὴν καρδίαν· ἐλάττονα δὲ οἱ τὸν τῆς κοιλίας στόμαχον ὑπολαβόντες ἐκ τῆς αὐτῆς οὐσίας ἀκριβῶς εἶναι τοῖς μυσίν. ἐγγύτερον μὲν γὰρ αὐτῶν ἐστι τῆς οὐσίας αὐτῆς ὁ ἔξωθεν χιτὼν, ὁ τὰς ἐγκαρσίας ἔχων ἶνας· οὐ μὴν οὐδ’ οὗτος ἀκριβὴς ὑπάρχει μῦς. Kühn maintains the manuscript reading ‘οἱ τὸν τῆς καρδίας στόμαχον’, which would make the discussion here not about the oesophagus, but about the ‘neck of the heart.’ Garofalo follows the Arabic translation and simply reads ‘τὸν στόμαχον’, however, a more conservative solution, which he offers in the apparatus and was adopted here, is to read ‘οἱ τὸν τῆς κοιλίας στόμαχον’ (‘neck of the stomach’), that is, the oesophagus. These texts not only give the impression that there were physicians who identified the heart and the stomach as muscles, but also that Galen acknowledged the *prima facie* reasonableness of such an identification on anatomical grounds.

Thus, Galen’s insistence that the heart and the stomach are not ‘strictly speaking’ (ἀκριβής) muscles raises the question of why he would maintain such a stance. If something resembles a muscle and moves like a muscle, it may be reasonable to classify it as such, rather than resorting to argue contrary to his own anatomical impressions. Additionally, if the heart and the stomach are not muscles, what are they?

## 3 Muscles and Natural Moving Organs

To understand which category of body parts other than muscles the heart and the stomach fall into and their relationship to muscles, the best place to begin is the following passage from *Natural Capacities*:

**T1** Every moving organ in the body possesses motion in accordance with the layout of the fibers. Look into it first in the muscles, if you want, whose fibers are most manifest, and their movements are visible due to their vehemence. After the muscles, go and observe that all the natural organs as well move in accordance with the fibers […] For just as in the muscles, motions come about when each fiber is stretched and pulled toward its source, it is the same way in the stomach.14*Nat. Fac*. III 8 (223,8 – 22 Helmreich = II 169 K.): ἑκάστῳ γὰρ τῶν κινουμένων ὀργάνων ἐν τοῖς σώμασι κατὰ τὰς τῶν ἰνῶν θέσεις αἱ κινήσεις εἰσίν. ἐπ᾿ αὐτῶν δὲ πρῶτον τῶν μυῶν, εἰ βούλει, βασάνισον τὸν λόγον, ἐφ᾿ ὧν καὶ αἱ ἶνες ἐναργέσταται καὶ αἱ κινήσεις αὐτῶν ὁρῶνται διὰ σφοδρότητα. μετὰ δὲ τοὺς μῦς ἐπὶ τὰ φυσικὰ τῶν ὀργάνων ἴθι καὶ πάντ᾿ ὄψει κατὰ τὰς ἶνας κινούμενα. … ὥσπερ γὰρ ἐν τοῖς μυσὶν ἑκάστης τῶν ἰνῶν τεινομένης τε καὶ πρὸς τὴν ἀρχὴν ἑλκομένης αἱ κινήσεις γίγνονται, κατὰ τὸν αὐτὸν λόγον κἀν τῇ γαστρί.

Here, Galen introduces a category, ‘moving organs,’ with two sub-groups: muscles, and Natural Moving Organs. Both species of moving organs are said to share basic anatomical and physiological features.

Fibers, as the anatomical facilitators of motion, and motion along the course of the fibers as their proper activity, constitute the fundamental principles unifying all moving organs in the body. The basic kinematic principles governing all moving organs, muscles and Natural Moving Organs alike, are the same, as they move by contraction (συστολή) and release (ἔκτασις) of their fibers.15In the first book of *The Motion of Muscles*, esp. *Mot. Musc*. I 5 (11 – 12 Rosa = IV 391 K.); I 8 (16 – 19 Rosa = IV 401 – 407 K.), Galen argues at length that, in the case of muscles, it is strictly speaking contraction and not extension that is the proper motion of muscular fibers. This is because contraction is the natural kinetic tendency of fibrous matter, while extension occurs either mechanically (when the antagonist muscles contract or when the anatomical layout of the muscles stretches) or through the external influence of the nerves on the muscles. According to various sources in Galen, and as glimpsed in his discussion of the stomach above, fibers come in three varieties: longitudinal (εὐθεῖα), transverse (ἐγκάρσια), and oblique (λοξή).16On the exact definition of these terms, see Singer 2023, 208, n. 20. In muscles, the arrangement of fibers determines the direction towards which they will contract and from which they will expand upon motion. In Natural Moving Organs, the same three types of fibers appear to serve different capacities: straight fibers aid in attracting the organ’s proper substance towards it by ‘pulling’ the adjacent anatomical structures in which it is contained toward it; oblique fibers facilitate the retention of appropriate substances within the natural organs; finally, transverse fibers allow expulsion by peristaltic motion.17*Nat. Fac*. III 8 (231 – 232 Helmreich = II 180 K.); III 11 (232 – 233 Helmreich = II 180 – 182 K.); III 13 (240,20 – 242,4 Helmreich = III 193 – 194 K.).

Although we learn from T1 which group of organs the stomach, heart, and other non-muscular moving organs belong to, it only further problematizes the very distinction it presents. According to T1, muscles and Natural Moving Organs are so similar in matters of anatomy and physiology, that observing the clearer relationship between muscular movements and the arrangement of muscular fibers teaches us about the analogous relationship obtained between Natural Moving Organs and their fibers.18Compare to a similar inference about Natural Moving Organs from muscles in *The Distinct Types of Uniform Parts* (*Part.Hom.Diff*. 9 1 – 2 [80,14 – 82,4 Strohmaier]). Now we can rephrase the question raised at the outset of the paper about the heart and the stomach in more general terms: if Natural Moving Organs and muscles are so similar, why distinguish between them in the first place?

The first, and most studied, ground for distinction on Galen’s part is also the most theoretical, namely his division of the soul. Galen famously adopted and expanded upon Plato’s tripartite division.19De Lacy 1988; Hankinson 1991; 2006; Vegetti 1999; Schiefsky 2012. He located the rational soul in the brain, the spirited soul in the heart, and the desiderative or nutritive soul in the liver. Following Stoic usage, he sometimes refers to the nutritive soul as ‘nature’ (φύσις), distinguishing it from ‘higher’ activities which are called soul more properly.20For the Stoic doctrine of the soul, see Inwood 2014, with references to sources and previous bibliography there. Galen insisted on the separateness of the soul parts, meaning that each of the three performed its activities by or inside itself as well as through its distinct transmission system.21*MM*. IX 10 (X 635 – 636 K.); *Ars*.5 (I 318 K.). In *PHP*. VI 2 (368,13 – 369,9 De Lacy = V 515 – 516 K.), Galen commends Plato for thinking about the soul as made up (συνκειμένη) of three distinct parts (μόρια) or forms (εἴδη) and criticizes Posidonius and Aristotle for thinking about the soul as one substance (οὐσία) with three powers (δυνάμεις). The brain is responsible for intellectual activities such as thought and memory as well as for sensation and voluntary motions through the nerves. The heart provides the body’s *tonos* and maintains its proper levels of natural heat through the arteries. The liver produces blood necessary for nutrition and is the source of the veins through which the blood flows to other body parts.22*PHP* VII 3.2 – 3 (438,28 – 440,8 De Lacy = V 600 – 601 K.).

In this schema, Galen categorizes the body’s capacities, the activities they enable, and the organs carrying them out into two types: natural (φυσικαί/φυσικά), which fall under the purview of the desiderative and spirited souls, and psychic (ψυχικαί/ψυχικά), which are the responsibility of the rational soul. Psychic capacities enable psychic activities, which are perception and voluntary motion (κίνησις καθ’ ὁρμήν). These are carried out by psychic organs, which are the organs of perception and the muscles. Natural capacities enable, in turn, natural activities, which include attraction, retention and expulsion. These are carried out by Natural Moving Organs when performing their functions of ferrying materials (food, *pneuma*, urine, foetus) through the body and keeping them stationary when necessary.23All organs, including muscles and Natural Immobile Organs have a form of the natural capacities of attraction, retention and expulsion which they require for their specific nourishment, but these activities do not involve spatial motion of these organs (see Lewis 2023, 289 – 292).^,^24*Nat.Fac*. I 1 (101,1 – 15 Helmreich = II 1 K.); *UP*. I 16 (I 32,24 – 33,15 Helmreich = IV 45 – 46 K.). On capacities in Galen, see Singer 1997; Hankinson 2014; 2024; Harari 2016. As Leith 2020, 32 – 8 shows, Herophilus already seems to have distinguished between voluntary and natural motile capacities. The term muscles, therefore, refers in Galen’s language solely to the ‘instruments of voluntary motion’ that exist to execute psychic activities.25*Mot.Musc*. I 1 (1,1 Rosa = IV 367 K.): ὀργανα κινήσεως τῆς καθ’ ὁρμήν οἱ μύες είσίν. See also *UP*. VI 18 (I 364,23 – 365,5 Helmreich = III 501 K.), where the heart is said to have a ‘natural action’ (φυσικὸν ἔργον) and to ‘rely on no psychic activity’ (μηδεμίαν ἐνέργειαν ψυχικὴν πεπιστευμένη), while muscles are ‘organs for a psychic activity’ (ψυχικῆς ἐνεργείας εἰσὶν ὄργανα). This criterion thus disqualifies the stomach and the heart from being muscles since their movement is evidently beyond our control and hence does not involve the rational soul. In short, while both Natural Moving Organs and muscles are moving organs, as stated in T1, the muscles are not natural, but rather psychic moving organs.26*Mot.Musc*. I 3 (5,20 – 25 Rosa = IV 377 K.); *Bon.Mal.Suc*. 4.18 – 9 (402 – 403 Helmreich = VI 771 – 772 K.); *Loc.Aff*. I 7 (302,16 – 304,2 Gärtner = VIII 66 – 67 K.).

Considered by itself, however, this explanation for the distinction between muscles and other moving organs in Galen’s taxonomy makes it appear as an external imposition. Were we to leave Galen’s reasons for the division at that, it would seem that he overlooks, underappreciates or even outright ignores the anatomical similarity between Natural Moving Organs and muscles in order to maintain his idiosyncratic preferences for Platonic psychology. On such an interpretation the distinction would also turn out quite uninformative, as it would not reveal anything of interest about Galen’s view of the body and its functions beyond the already known fact that he adhered to Platonic tripartition. However, as we shall argue, the situation is quite different. While Galen’s broader theoretical commitments undoubtedly influenced this typology of bodily organs, we argue that he fundamentally grounds this typology in perceived anatomical, and more specifically morphological, differences among types of organs.27Morphological anatomical differences refer to the structure and spatial arrangement of the organs and their parts; qualitative anatomical differences refer to their perceptible qualities, such as colour, texture, taste, etc. These anatomical distinctions, in Galen’s view, serve as the foundation for the physiological differences he observes, thereby supporting and substantiating his philosophical and psychological framework.

## 4 Anatomical Differences

Although both muscles and Natural Moving Organs are composed of motion-facilitating fibers and their own flesh, Galen detects anatomical differences between the two types of organs. These concern primarily the origin and layout of the organs’ fibers. A passage in which Galen attempts to differentiate between the heart and the muscles illustrates some of these differences.

**T2** The heart is composed of fibers varied in position, with simple flesh growing around each of them. This is, however, common to all muscles, as well as to the stomach, the intestines, the bladder, and the uterus. But neither do the fibers have the same thickness in terms of strength in all of them, nor does the flesh have exactly the same form. Instead, the flesh in the muscles is redder and more tender than that of the stomach, the uterus, the bladder, and all the intestines. The heart, however, is rougher and has a more varied layout of fibers. Muscles have a simple layout of fibers, but not so the heart, and similarly neither the proper tunics of the uterus nor those of the bladder.28*AA*. VII 8 (II 431,18 – 29 Garofalo = II 609 – 610 K.): τὴν μὲν καρδίαν ἐξ ἰνῶν ποικίλων τῇ θέσει συγκεῖσθαι λέγοντα, περιφυομένης αὐτῶν ἑκάστῃ σαρκὸς ἁπλᾶς. κοινὸν γὰρ τοῦτο καὶ τοῖς μυσὶν ἅπασι, καὶ τῇ γαστρὶ, καὶ τοῖς ἐντέροις, κύστει τε καὶ μήτρα. οὐ μὴν οὔθ’ αἱ ἶνες ἴσην ἰσχὺν ἡ πάχος ἔχουσιν ἐν ἅπασιν, οὔτε ἡ σὰρξ τὴν αὐτὴν ἀκριβῶς ἰδέαν. ἀλλ’ ἡ μὲν ἐν τοῖς μυσὶν ἐρυθροτέρα τ’ ἐστὶ καὶ μαλακωτέρα τῆς κατὰ τὴν γαστέρα, καὶ μήτραν, καὶ κύστιν, ἅπασάν τε τὴν τῶν ἐντέρων οὐσίαν· ἡ δὲ τῆς καρδίας σκληροτέρα ἅμα καὶ ποικιλωτέρα ταῖς ἰσίν. ἁπλῶς μὲν γὰρ ἔχουσι τῇ θέσει τὰς ἶνας οἱ μύες, οὐχ ἁπλῶς δὲ ἡ καρδία, καθάπερ οὐδ’ ὁ τῆς μήτρας ἴδιος χιτὼν, οὐδ’ ὁ τῆς κύστεως.

The most immediate differences between muscles and Natural Moving Organs are their differing textures and colors.29On colors in ancient Greco-Roman anatomy, see Vespa and Lewis Forthcoming. In this regard, according to Galen, all the muscles share basic qualitative similarities: the subtle and small extraocular muscles that support the eye in its motions and the large and bulky gluteus maximus are made of the same kind of material, while the stomach and the bladder are not. Later in the same paragraph he also describes the contrast in flavor between muscles and Natural Moving Organs, especially the heart, as an additional indication of their distinct material constitution.30*AA*.VII 8 (II 433.21 – 28 Garofalo = II 611 – 612 K.). See also *Temp*. III 3 (58,8–14 Helmreich = I 601 K.). The similarity Galen finds between all muscles makes him insist that the term ‘flesh’ (σάρξ) in its strict sense can only be applied to muscles, namely to their material substance. The different substances of Natural Moving Organs are all too dissimilar to muscle to be counted as ‘flesh’ as well. He does, however, allow the application of the term to the material of such parts, due to a lack of a better word.31*MM*. X 11 (X 730,16 – 731.2 K.).

Beyond the basic sensory distinctions by touch and sight, T2 conveys an additional morphological differentiation regarding the arrangement of fibers in these organs. Galen thinks that muscles have a simple fiber arrangement: each muscle has only one kind of fiber from the three mentioned above, that is, its fibers run in one direction only.32In *AA*. I 3 (I 15,15 – 20 Garofalo = II 228 K.), Galen distinguishes between muscles with a ‘simple’ (ἁπλῆ) fiber layout and those with a double (διττή), more variegated (ποικιλώτερα) and crisscrossed (ἔμπαλιν) layout. Even in these cases, however, each muscular layer has fibers with only one direction, unlike Natural Moving Organs. In contrast, the heart, uterus and bladder have a variegated fiber layout, in which straight, transverse, and oblique fibers are interwoven. From other sources, we learn that all Natural Moving Organs either have more than one type of fiber in their tunic or multiple layers of tunics.33*Nat. Fac*. III 11 (231,19 – 232,10 Helmreich = II 180 – 181 K.). Sometimes Galen seems to say that intestines are the only exception to this rule, performing only the expulsive capacity and thus having only transverse fibers; see, e.g., *UP*. V 10 (I 282,12 – 9 Helmreich = III 385 – 386 K.). However, in his definite account in *Anatomical Procedures* he clarifies that while they have mostly (πλεῖσται) transverse fibers (here called circular, κυκλοτερεῖς), they also have other fibers, see *AA*. VI 7 (II 381,29 – 30 Garofalo = II 569 K.).

A related and key difference between muscles and Natural Moving Organs pertains to the source of the fibers in muscles and other organs. This idea is most fully developed in *The Motion of Muscles*, where we learn that the fibers of muscles are actually small nerve and ligament filaments infiltrating the muscle flesh and intermingling with it.

**T3** The nerves are similar to channels, conveying the capacities from the brain as from a certain source to the muscles. When first it [sc. the nerve] enters them [sc. muscles], it splits in various ways, one division upon another, and at last is resolved into thin and membrane-like fibers so that the entire body of the muscle interweaves with them.34Images of such branching of nerves inside are available on https://dissections.atlomy.com/nerve-fibres-in-muscles-14 (Lewis 2024) (last accessed December 20, 2024). Note that the images are captures from dissections (approved by the EU strict ethics regulations). The ligaments, by which the muscles are bound and grow together with the bones, generate the membranes around them [sc. muscles], and send some inward branches into the flesh of the muscles themselves.35*Mot.Musc*. I 1 (3,3 – 9 Rosa = IV 371 – 372 K.): λόγον οὖν ὀχετῶν ἔχοντα τὰ νεῦρα καθάπερ ἔκ τινος πηγῆς τοῦ ἐγκεφάλου τοῖς μυσὶ παράγονται τὰς δυνάμεις. ἐπειδὰν πρῶτον δ’ αὐτοῖς ὁμιλήσῃ, παράγονται τὰς δυνάμεις. ἐπειδὰν πρῶτον δ’ αὐτοῖς ὁμιλήσῃ, σχίζεται πολυειδῶς ἄλλην ἐπ’ ἄλλῃ σχίσιν, καὶ τέλος εἰς λεπτὰς καὶ ὑμενώδεις ἶνας ὅλα λυθέντα πᾶν οὕτω τὸ τοῦ μυὸς σῶμα διαπλέκει. οἱ δ’ αὖ σύνδεσμοι, καθ’ οὓς τοῖς ὀστοῖς οἱ μύες συνδοῦνται καὶ συμφύονται, τούς θ’ ὑμένας τοὺς ἀμφ’ αὐτοὺς γεννῶσι, καί τινας εἴσω διαφύσεις εἰς αὐτὴν τὴν σάρκα τῶν μυῶν πέμπουσιν. See also *Mot. Musc*. I 2 (4 Rosa = IV 375 K.); *AA*. IV 11 (I 265,10 – 13 Garofalo = II 473 K.).

According to Galen, muscle fibers are not made out of muscle material at all. In fact, as he explicitly writes in *The Distinct Types of Uniform Parts*, only flesh, and not fibers, is ‘the actual body of the muscle, peculiar to it’ (*jarmu l-ʿuḍlah al-khāṣṣī bihā*).36*Part. Hom. Diff*. 9 2 (82,4 Strohmaier): جرم العضلة الخاصى بها. The treatise survives only in ʿĪsā ibn Yaḥyā’s ninth-century Arabic translation (probably from Syriac). This is so because muscle fibers come from outside as nerves and ligaments that gradually differentiate themselves and branch off inside the flesh into minute fibrous strands ‘thinner than the strands of a spider web’ (ἀραχνῶν λεπτοτέρας).37*Sem*. I 10 (102,17 – 19 De Lacy = IV 551 K.). When properly defined, the muscle’s own substance is just this interfibrous flesh mass.38See the discussion in Meyer-Steineg 1911, 177 – 178. This conceptualization has practical implications for anatomical study; Galen advises observers to pay close attention (προσεχεῖν νοῦν) to the layout of muscle fibers (θέσις τῶν ἰνῶν τοῦ μυός) during dissection, as their directionality guides (ποδηγοῦσι) one to the insertion points of the original nerves from which they branch.39*AA*. IV 2 (I 219,6 – 9 Garofalo = II 426 K.).

Galen distinguishes this muscular arrangement from that of Natural Moving Organs, such as the heart and stomach. He posits that Natural Moving Organs possess a ‘different kind of fibre’ (ἕτερος γένος ἰνῶν) than muscles, which is specific or proper (ἴδιον) to them.40*Temp*. II 3 (58,25 – 26 Helmreich = II 602 K.). While the adjective ἴδιον can denote material differences in fiber composition, the context suggests it primarily refers to their origin. Galen contrasts muscle fibers, which he considers ‘parts of the nerves and ligaments’ (νεύρων καὶ συνδέσμων μόρια), with the ‘proper kind of fibers’ (τῶν ἰνῶν γένος ἴδιον) found in Natural Moving Organs. The latter are deemed proper in that they do not originate from external sources, namely nerves and ligaments. Rather, they are inherent to the Natural Moving Organs and form their actual physical constitution. Such organs are different from muscles not only in the substance from which they are made – being a different kind of uniform part – but also in their fibers, both in terms of layout and anatomical origin.41Throughout the whole of *The Distinct Types of Uniform Parts* Galen seems to treat fibers as a *sui generis* uniform part, implying that both Natural Moving Organ fiber and muscle fibers are, ultimately, of the same substance. Furthermore, in *Semen* (*Sem*. I 10 [102,13 – 16 De Lacy = IV 551 K.]), he proposes that both types of fibers originate from semen, the same substance from which nerves and ligaments derive. This common generative origin suggests that ἴδιον in the passage from *Mixtures* primarily refers to the fibers’ anatomical origin rather than their fundamental constitution.

A third morphological difference is the relatively straightforward observation that Natural Moving Organs are hollow while muscles are bulks of mass. Galen’s most direct engagement with this issue arises when he reflects on the term used to describe the walls of Natural Moving Organs, their tunics (χιτῶνας). According to Galen, this is a rather unusual (οὐ πάνυ τι κυρίως) application of the term since tunics are supposed to cover something else.42*AA*. VI 7 (II 381,12 – 25 Garofalo = II 568 K.); *AA*. X 9 (79,14 – 80,5 Simon). This is the case, Galen tells us, with the tunics that cover the eye and the tongue. However, in the case of Natural Moving Organs, the tunics are themselves the bodies that constitute the organ, and they have no ‘other substance’ (*jawhar ākhar*) besides their coverings.

Thus far, we have identified three pertinent anatomical differences between Natural Moving Organs and muscles: (1) muscles are invested with fibers from the nerves, while Natural Moving Organs have their own unique fibers; (2) muscles and Natural Moving Organs have different fiber arrangements, with that of Natural Moving Organs being more variegated and (3) Natural Moving Organs are hollow organs, with only coverings or tunics for bodies, while muscles are bulks (see Table 2). These are not mere morphological differences, for Galen they have functional significance which further evinces the distinction under discussion.

**Table 2:** Anatomical Comparison. A comparison between Muscles and Natural-Moving Organs in terms of anatomical properties.

	Muscles	Natural Moving Organs
Origin of fibers	Externally invested from the nerves and ligaments	Internal constituents of the organ mass.
Fiber layout	Simple, each muscle layer has one kind of fiber.	Complex, each Natural Moving Organ has all three fiber kinds.
Structure	Muscles are bulks of flesh	Natural Moving Organs are hollow

## 5 Physiological Differences

The first anatomical difference between Natural Moving Organs and muscles (Table 2) comfortably supports two interrelated physiological distinctions: first, muscles are moved from outside by the nerves, while Natural Moving Organs move themselves; second, the motile capacities of muscles are centrally managed by the brain, while the motile capacities of Natural Moving Organs are diffused throughout the body, with each Natural Moving Organ having its own principle of motion. All muscular movement is coordinated by the brain through an interconnected system of motor nerves emanating from the brain and spinal cord. Although there are ambiguities as to how exactly this occurs, one may generally say that commands of voluntary motion pass from the brain to the nerves through changes in the interconnected system of brain ventricles, psychic *pneuma* and nerves.43Galen presents three alternatives to the possible ways in which the *pneuma* in the brain activates motor and sensory nerves (*PHP*. VII 4 1 – 2 [448,4 – 18 De Lacy = V 611 – 612 K.]): (1) the nerves and the brain naturally contain pneuma, which is mechanically struck or plucked (πληττόμενον) by the brain to move the nerves and thus cause motion; (2) the nerves are usually empty of *pneuma* and it flows (ἐπιρρεῖ) into them from its reservoir in the brain to cause motion; (3) a qualitative change (ἀλλοίωσσις) in the pneuma inside the brain is transferred to the brain-matter inside the nerves without any change in the distribution of *pneuma* between them. This is possible because the substance of the brain and the substance of the internal core of most nerves form one, contiguous body (συνεχὲς σῶμα). The question of neural transmission vexed Galen; for some discussion see Kupreeva 2004; Singer 2020. Thus, it is not so much the muscle flesh itself, but rather the nervous fibers around which this flesh is woven that react to the motor directions and capacities from the rational soul. Although the flesh of the muscle is of the right kind to be moved by the nerves through the brain, being elastic and contractable, when severed from the nervous sources of motile capacities, it is inert and does not respond to stimuli.44The same is true for the tendons at the extremities of muscles, which are formed from nervous and ligamental fibers as well, see *UP*. I 17 (I 33,25 – 34,24 Helmreich = III 47 K.). All muscle fibers, therefore, form one interconnected nervous system centered in the brain, their motile power ‘flowing into them from a great source above, since they do not have it from themselves nor is it innate to them.’45*Mot.Musc*. I 1 (2,24 – 25 Rosa = IV 371 K.): ἄνωθεν ἀπὸ τῆς μεγάλης ἀρχῆς ἐπιρρεουσα ·οὐ γὰρ δὴ ἐξ αὑτῶν γ’ οὐδὲ σύμφυτον αὐτὴν ἔχει.

In contrast, every natural organ is endowed with exactly the kind of capacity that muscles lack, an innate (σύμφυτος or ἕμφυτος) capacity which is its proper principle of motion and rest, allowing it to execute its activities without the involvement of an outside moving force, such as the one coming from the brain: ‘natural organs differ in this regard very much from psychic organs, since it has been shown that the capacity for an activity is innate in natural organs, but flows into psychic organs from a source like light rays from the sun.’46*Nat.Fac*. III 5 (I 215,6 – 9 Helmreich = II 157 K.); *MMG*. II 4 (XI 96 – 97 K.); *Caus.Symp*. III 5 4 (VII 237,1 – 3 K.); *Prop.Plac*. 9 3 (91 Garofalo\Lami). For an analysis of what Galen means by the claim that natural organs move themselves and the difference between his claim and earlier, especially Peripatetic, accounts of the causal analysis of bodily changes and motions, see Harari 2016. The anatomical origin of fibers dovetails with the origin of the motion they facilitate, externally induced motion for the muscles and internally instigated for Natural Moving Organs.

The second anatomical distinction also grounds an important physiological difference: Natural Moving Organs require all three types of fibers because each type facilitates a different activity the organ performs, attraction, retention, and expulsion.47*Nat. Fac*. III 11 (231,19 – 232,10 Helmreich = II 180 – 181 K.). In contrast, each muscle is designed to perform a single contracting motion, so it has a single type of fiber. In *The Motion of Muscles*, Galen engages in a long argument against the competing opinion that muscles, such as those supporting the tongue, engage in more than one motion. He attributes this disagreement to his opponents’ anatomical ignorance, asserting that the tongue muscle is composed of six different muscles, each having only one activity in the grand scheme of supporting the varied operations of the tongue.48*Mot.Musc*. I 4 (7,27 – 10,13 Rosa = IV 382 – 387 K.) and see note 13 above. Conversely, the stomach, as we have seen, engages in multiple motions as part of its proper activities. Similarly, in *The Function of the Pulse* (*Us.Puls*. 6 [214,31 – 218,25 Furley\Wilkie = V 169 – 172 K.]), Galen argues that arteries have both contraction and expansion as their proper activities, because they are endowed with the capacity to actively alternate between them.

Finally, the last anatomical difference also serves as a basis for the differentiation of the activities in which muscles and Natural Moving Organs engage. The role of the latter is to transport and house substances: the stomach and intestines move food from the mouth to its more substantial elaboration into blood inside the liver, the uterus houses and transports the foetus, the bladder urine, and the heart *pneuma* and pneumatized blood. Importantly, no moving organ is specifically responsible for the elaboration or transformation of substances, a role that, as we shall argue below, Galen usually reserves for another category of organs, Natural Immobile Organs.49For the possible exception of the stomach, see p. 23 below. Natural Moving Organs serve as directing mechanisms to regulate the economy of substance flow in the body. Naturally, the form of such organs is that of a hollow organ or a cavity. This is why, when describing the formation of the intestines and stomach, Galen singles out ‘the shape of the internal cavity’ (τὴν τῆς ἔνδον κοιλότητος ἰδέαν) as one of the important features of these organs’ teleological design.50*Nat.Fac*. I 6 (111,23 – 24 Helmreich = II 15 K.). Conversely, muscles exist for the sake of motions, needing no cavity in them.

We see, therefore, how close anatomical observations, physiological explanations and philosophical commitments are enmeshed in Galen’s insistence on the difference between muscles and Natural Moving Organs (see Table 3). Although both share broad anatomical and physiological similarities, being motile and fibrous, they differ in their anatomical characteristics and the subsequent physiology this anatomy facilitates. This differentiation precludes their grouping as identical organ types. Moreover, the relationship between anatomical, physiological and philosophical considerations extends beyond mere interconnectedness. Although it is difficult, as always, to determine the exact order of Galen’s research and analysis, it appears that the anatomical distinctions Galen deemed salient between the different organs played a foundational and substantiating role in establishing the physiological distinctions between these organ categories. This anatomical and physiological groundwork, in turn, supported and reinforced the psychological distinctions built upon it.51A small caveat is in place about the designation of the heart as a Natural Moving Organ. On the one hand, the heart is a Natural Moving Organ, a motile organ that participates in the natural activities of the organism by its fiber-induced motions. See, for example, *Symp.Diff*. 4 1 – 2 (228,1 – 17 Gundert = VII 62 – 63 K.) where the pulse is numbered as a natural activity and *UP*. VI 18 (I 364,23 – 365,5 Helmreich = III 501 K.) where the heart is said to have a ‘natural action’ (φυσικὸν ἔργον). As we have seen, it is often discussed in tandem with other Natural Moving Organs and contrasted with muscles. On the other hand, in other contexts and especially when the discussion is more theoretical and centers on the tripartition of the soul, the heart is singled out as a *sui generis* organ, the seat of the spirited soul and the origin of an independent system of vessels, the arteries, conveying their unique kind of substance, vital *pneuma* (πνεῦμα ζωϊκόν). Relatedly, the arteries’ motile capacity reaches them from the heart (*Art.Sang*. 8.2 [178,1 – 7 Furley\Wilkie = IV 732 K.]). Viewed from that perspective the heart is conceived as a separate source of capacities, on par with the liver and the brain, see, e.g., *Caus.Symp*. III 5 4 (VII 237,1 – 3 K.). These issues cannot be developed here.

**Table 3:** Anatomical and Physiological Comparison. A comparison between Muscles and Natural Moving Organs in terms of the interrelated anatomical and physiological properties.

Difference		Natural Moving Organs	Muscles
Fiber origin	Anatomy	Internal origin of fibers	External origin of fibers
	Physiology	Internal and diffused origin of motion	External and centralized origin of motion
Fiber layout	Anatomy	Complex: All three kinds of fibers	Simple: one kind of fiber
	Physiology	Complex motions according to direction of fibers	Simple, one-directional motion
Morphology	Anatomy	Hollow organs with tunics	Bulks
	Physiology	Designed to house and transport substances	Designed to facilitate voluntary motion

## 6 Natural Immobile Organs

T1 refers only to a subset of organs, namely moving ones. An anatomical common ground between all these organs is the existence of fibers that facilitate and determine their motions. In some places, when discussing the differences between muscles and Natural Moving Organs, Galen includes a third type of organ, which we label Natural Immobile Organs. These organs are natural and not psychic because they carry out natural activities, similar to Natural Moving Organs. However, their substance is without fibers and thus, unlike Natural Moving Organs, their activities do not involve perceptible movement.52Respiration involves, of course, a perceptible motion of the lungs. But, in Galen’s view, the lungs do not move themselves but are moved by the intercostal muscles. We thank Andrés Pelavski and an anonymous reviewer for this point. Consider, for example, this passage from *Mixtures*:

**T4** The flesh of the liver, the spleen, the kidneys, and the lung is simple in nature, sprouting around the arteries, veins, and nerves in each of the viscera. The form of the flesh of the heart, however, is not simple, but just as there are fibers in the muscles, to which the flesh is attached, such are in the heart too. However, the fibers are not of the same kind, but those in the muscles are parts of nerves and ligaments. But the kind of fibers of the heart is peculiar, just as the coat of the artery and of the vein and of the intestines, stomach, uterus, and of both bladders. For in all these organs, it is possible to see their own flesh attached to their own fibers.53*Temp*. II 3 (58,18 – 59,2 Helmreich = I 601 – 602 K.): ἀλλ’ ἡ μὲν τοῦ ἥπατός τε καὶ τοῦ σπληνὸς καὶ τῶν νεφρῶν καὶ τοῦ πνεύμονος σὰρξ ἁπλῆ τὴν φύσιν ἐστὶ ταῖς καθ’ ἕκαστον σπλάγχνον ἀρτηρίαις καὶ φλεψὶ καὶ νεύροις περιπεφυκυῖα. τῆς καρδίας δ’ οὐχ ἁπλοῦν τὸ τῆς σαρκὸς εἶδος, ἀλλ’ οἷαίπερ αἱ ἐν τοῖς μυσὶν ἶνές εἰσιν, αἷς ἡ σὰρξ περιπέπηγε, τοιαῦται κἀν τῇ καρδίᾳ. πλὴν οὐ ταὐτὸν γένος τῶν ἰνῶν, ἀλλ’ αἱ μὲν ἐν τοῖς μυσὶν νεύρων εἰσὶ καὶ συνδέσμων μόρια. τῆς καρδίας δὲ τὸ τῶν ἰνῶν γένος ἴδιον ὥσπερ καὶ τοῦ τῆς ἀρτηρίας τε καὶ τῆς φλεβὸς χιτῶνος ἐντέρων τε καὶ γαστρὸς καὶ μήτρας καὶ τῶν κύστεων ἑκατέρων. ἔστι γὰρ οὖν δὴ κἀν τούτοις ἅπασι τοῖς ὀργάνοις τὴν οἰκείαν σάρκα περιπεπηγυῖαν ἰδεῖν ταῖς ἰδίαις αὐτῶν ἰσίν.

Here, Galen explicitly presents the liver, kidneys, lungs, and spleen as a distinct category of internal organs or viscera (σπλάγχνα), for, unlike both Natural Moving Organs and muscles, their flesh is simple (ἁπλῆ) with no fibers mixed in it. All three groups of organs – muscles, Natural Moving Organs, and Natural Immobile Organs – are on full display in this short segment, and Galen carefully distinguishes between them based on criteria we saw earlier, the existence and layout of fibers.54The complex relationship between Natural Mobile Organs and Natural Immobile Organs and the other activity of rational souls, sensation, as well as the existence and role of sensory nerves in these organs calls for a separate examination. It seems that Natural Mobile Organs are endowed with more sensory nerves than Natural Immobile Organs, allowing the body to feel changes and disturbances in the former and not in the latter. See, e.g., *Plen*. 4 (VII 530,7 – 532,15 K.); *Nat.Fac*. III 12 (I 223,10 – 236,11 Helmreich = 182 – 186 K.); *UP*. IV 13 (I 226,2 – 232,9 Helmreich = III 308 – 311 K.).

In another passage from *Good and Bad Fluids*, where three groups of organs are juxtaposed again, Galen uses an interesting image to describe the simple flesh of Natural Immobile Organs relative to the complex features of muscles and Natural Moving Organs: ‘In these [sc. Natural Immobile Organs], there appears to be a substance like blood that has solidified between the vessels.’55*Bon.Mal.Suc*. 4 18 (402,13 – 14 Helmreich = VI 771 K.): φαίνεται γὰρ ἐπ’ ἐκείνων μὲν ἐν τῷ μεταξὺ τῶν ἀγγείων οὐσία τιϲ αἵματι πεπηγότι παραπλήσιος. Elsewhere, Galen compares the substance of Natural Immobile Organs, such as the lungs and liver, to a soft ‘filling’ (στοιβή) between the veins and arteries.56Liver: *AA*. VI 11 (II 391,15 Garofalo = II 576 K.); *Foet.Form*. 3 20 (70,20 – 22 Nickel = IV 668 K.); Lungs: *UP*. VII 1 (I 376,1 Helmreich = III 517 K.). As discussed earlier, Galen grapples with the appropriate term to describe the substance between the fibers of Natural Moving Organs. While he denies it the status of flesh, he allows its analogical application due to the absence of a more suitable term. The other common term, ‘tunic,’ also has its problems. A similar phenomenon occurs here when Galen attempts to describe the substance constituting Natural Immobile Organs. Referring to the substance of Natural Immobile Organs as a filling or solidified blood between the vessels echoes another term commonly used to describe the same solid components, particularly by Erasistratus and his followers, namely *parenchyma* (παρέγχυμα). This term, a result noun of the verb παρεγχέω, ‘to pour alongside,’ was employed by Erasistrateans to refer to the solid parts between the nerves, arteries, and veins. It most likely alluded to their formation resulting from the ‘pouring over’ of nutriment, that is, blood, from the surrounding vessels and their subsequent solidification.57For the etymology, see *SMT*. XI 1 (XII 311,2 – 7 K.). For a more comprehensive discussion of their role in Erasistratus, see Leith 2015.

Galen’s stance on the term *parenchyma* is somewhat ambivalent. On the one hand, he rejects it because it implies that the formation of vital organs, such as the liver and lungs, is, to some extent, accidental, resulting from a pour-over from the vessels.58*Temp*. I 3 (57,10 – 15 Helmreich = I 599 – 600 K.); *Comp.Med.Loc*. VIII 6 (XIII 192,18 – 193,1 K.). On the other hand, this term provides Galen with a convenient word to describe the substance of these organs without using the term flesh, which, as demonstrated earlier, he reserves specifically for muscles. Thus, when discussing the liver, lungs, and spleen, Galen presents *parenchyma* as a possible option to describe their ‘flesh’, without committing to its otherwise problematic literal etymology.59Liver: *AA*. VI 11 (II 391,13 – 14 Garofalo = II 576 K.); *PHP*. VI 8 (412,1 – 2 De Lacy = V 561 K.); Lungs: *UP*. VI 4, (I 307,15 – 17 Helmreich = III 421 K.); *AA*. VII 5 (II 423,16 – 18 Garofalo = II 603 K.); Spleen: *UP*. IV 15 (I 233,15 – 16 Helmreich = II 318 K.).

Since fibers are the *sine qua non* of bodily motion, the fibreless constitution of Natural Immobile Organs means that they execute natural capacities like Natural Moving Organs, but without moving themselves in the process. At the risk of oversimplification, Galen’s view of Natural Moving Organs activity involves significant mechanical components that Natural Immobile Organs inherently lack.60The term mechanical is fraught with problems, see Berryman 2002. Our use of the term mechanical follows Lonie’s (1981, 123) second definition of the term as a change explained ‘in terms of the mutual contact and pressure of bodies, both solid and fluid’. Such ‘pressure’ or contact is exactly what Natural Immobile Organs do not need to engage in their activities. For instance, as previously discussed, Galen’s explanation of stomach operation revolves around its mechanical motions: it attracts food by pulling the oesophagus along its longitudinal fibers, contracts around the food using oblique fibers, and excretes it through transverse fibers. When Galen describes the operations of Natural Immobile Organs, on the other hand, he never refers to any mechanical motion on their part. They attract without pulling, retain without contracting, and expel without pushing. For instance, in his discussion of the spleen, Galen emphasizes that the very substance of the organ, its *parenchyma*, is what allows it to attract black bile into itself, seemingly without moving itself or any of the parts to which it is connected.61Notably, these two modes of attraction differ from another division Galen makes between attraction ‘by the filling of void’ (τὸ τῇ πρὸς τὸ κενούμενον ἀκολουθίᾳ) and ‘by appropriateness of quality’ (τὸ οἰκειότητι ποιότητος). Since both the stomach and the spleen discriminate between different substances, their attractions are instances of the latter category. The difference between them lies in the amount of mechanical labor involved in the execution of the process. See Berryman 2002; Adamson 2014; Lewis 2023, 288 –289 for further discussion.

The most critical activity of Natural Immobile Organs, however, is to qualitatively transform incoming substances in ways that Natural Mobile Organs cannot. They accomplish this transformation without perceptible motion, but rather by the unique mixture of their substance. This is true of all the organs listed in T4: the flesh of the kidneys transforms the serous parts of blood into urine; the spleen similarly attracts black bile; the lungs elaborate air into *pneuma* and the liver food into blood.62For the lungs, see Rocca 2020; for the kidneys, Temkin 1961; Scarborough 1976; for the spleen, Stewart 2019, 104 – 128. We will briefly consider as an example the anatomy and physiology of the liver, whose very flesh Galen designates as ‘the primary tool for making blood’.63*UP*. IV 13, (I 222,16 Helmreich = III 303 K.): σάρκα τοῦ ἥπατος … τὸ πρῶτον τῆς αἱματώσεως ὄργανον.

The liver receives food from the gastrointestinal tract to the portal vein (ἡ ἐπὶ πύλαις φλέψ) on its concave side, this food then undergoes a series of ramifications.64Four large veins from the gastrointestinal tract merge into the portal vein. These can be roughly identified with the right gastric vein, the splenic vein (which branches into the left gastric vein), the inferior mesenteric vein, and the right gastroepiploic vein; see *AA*. XIII 2 (179,10 – 180,8 Simon).

**T5** You will find from each lobe of the liver, how many they may be, one penetrating vessel divided into many small ones like a tree trunk into branches [which are] again divided into something like twigs and terminating in something like thin shoots. Whatever space there is between the vessels, all of it is filled with the flesh of the viscus.65*AA*. VI 11 (II 391,9 – 13 Garofalo = II 576 K.): ἀπὸ γὰρ τῶν πυλῶν εἰς ἕκαστον λοβὸν, ὅσοι περ ἂν ὦσι, μίαν εὑρήσεις ἀφικνουμένην μεγάλην φλέβα, ἧς σχισθείσης εἰς πολλὰς μικρὰς, ὥσπερ στέλεχος εἰς κλάδους, εἶτ’ αὖθις ἐκείνων οἷον εἰς ἀκρέμονάς τινας σχιζομένων, εἶτ’ ἐκείνων ὥσπερ εἰς βλαστήματά τινα λεπτὰ τελευτώντων, ὅσον ἐστὶ μεταξὺ τῶν ἀγγείων, ἅπαν πεπλήρωται τῇ τοῦ σπλάγχνου σαρκί. See also *AA*. XIII 2 (181 – 182 Simon); *Ven.Art.Diss*. 1 30 (82,4 – 13 Garofalo\Debru = II 785 K.). The arboreal analogy of vessels to roots, trunk and branches appears quite often in Galen, see, e.g., *Ven.Art.Diss*. 1 1 – 5 (76,9 – 77,8 Garofalo\Debru = II 780 – 781 K.).

Thus, the portal vein is divided with a single branch going into each lobe of the liver. As these branches penetrate deeper into the liver’s flesh, they ramify further into increasingly smaller vessels with exceedingly thinner tunics. Galen teleologically justifies this meandering vascular labyrinth and what he perceives to be the extremely thin walls of the veins inside the liver by the fact that this arrangement allows the food to gain maximum exposure to the liver’s flesh, facilitating thorough sanguification.66*UP*. IV 13 (I 224 Helmreich = III 30 K.).

From the convex side of the liver, blood is taken by the hollow vein (*koilē phleps*, namely the inferior vena cava) and distributed to the rest of the body. Similar to the portal vein, Galen likens the source of the vena cava to a tree:

**T6** Its origin is not solely from a specific location on the convexity of the liver; rather, it originates from all the veins within the liver’s convexity, as if it gathers and merges from its roots, ascending like a tree trunk divided into two parts, one ascending towards the diaphragm, and the other descending towards the kidneys.67*AA *. XIII 3 (183,8 – 11 Simon): منشاه ايضا من موضع واحد من حدبة الكبد بل منشاه من جميع العروق التى فى حدبة الكبد كانه يجتمع ويلتام من اصول له ويرتقى مصعدا بمنزلة ساق شجرة مقسوم باثنين وأحد جزويه يرتقى الى الحجاب والجزء الاخر ينحدر الى الكليتين.

Interestingly, however, Galen does not mention a vascular connection between the ‘branches’ of the portal vein and the ‘roots’ of the vena cava. In *The Function of the Parts of the Human Body*, he hints that these vessels ‘are not connected to each other’ (οὐ συνάπτονται) for a specific physiological purpose, which he, unfortunately, does not explicate.68*UP*. XII 13 (I 222,14 – 5 Helmreich = III 301 K). He sets the question ‘why are the veins in the convex part not connected to the veins in the concave part?’ (διὰ τί ταῖς ἐν τοῖς σιμοῖς φλεψὶν αἱ ἐν τοῖς κυρτοῖς οὐ συνάπτονται) as one of the components of a teleological understanding of the liver. Unlike other questions presented in the same pericope, he does not address it afterwards. In *Affected Places*, he adds that although the connections (συναναστόμοσεις) between the two vessels inside the liver are not detectable, nonetheless, there is unanimous agreement that food somehow passes from the extremities (πέρας) of the thin branches of the portal vein to the extremities of the vena cava and is then distributed (ἀναδιδομένην) to the rest of the body.69*Loc.Aff*. V 7 15 (344,15 – 22 Brunschön = VIII 351 – 352 K.).

From this description, it appears that blood passes through the actual flesh of the liver on its way from one set of vessels to the next. Thus, digested food is not only exposed to the unique flesh of the liver inside extremely thin veins but also directly, as it passes through the ‘hepatic gap’ between the offshoots of the portal vein and the origins of the vena cava. Given the fact that Galen attributes a function to this gap, it seems reasonable to conclude that, similarly to the convoluted route and the thin walls inside the liver, it serves to enhance the exposure of blood to the liver before its distribution to the rest of the body from the vena cava.70May (1968, 1.54) suggests that Galen’s point is that although vascular passages between the two sets of veins are unseen, he assumes that they nonetheless exist. However, in *Affected Places*, Galen is only committed to the claim that blood passes from one set of veins to the other and not to the claim that it passes through an additional set of invisible vessels. Given his explicit statement in *The Function of the Parts of the Human Body* that the vessels are not connected, it seems more appropriate to interpret him as meaning that food passes without any continuity between the vessels. While passing through the flesh of the liver, the food finally turns to blood and is purified from black and yellow bile, which flow to the spleen and the gallbladder respectively.71On these two substances in Galen and in the tradition that he inherited, see Nutton 2005, Soleil 2011, Jouanna 2012, Ezrokhi 2023.

To conclude, there is a third category of organs often mentioned alongside Natural Moving Organs and muscles. Their substance is simple and fiber-less and their activities, subsequently, do not involve perceptible movements. They share important similarities with Natural Mobile Organs, both being natural organs: each Natural Immobile Organ is constituted from a unique material blend that allows it to fulfill its specific role in the body. However, their roles tend to center more on the transformation and less on the active transportation of substances and be carried out without moving. Since motion is unnecessary for Natural Immobile Organs, nature did not endow them with fibers to facilitate it.

## 7 Conclusions

Galen’s unique tripartition of internal bodily organs into muscles, Natural Moving Organs, and Natural Immobile Organs elucidates the initial query of this paper – why the heart and stomach (along with other muscle-like organs) are not classified as muscles. This tripartite distinction is fully displayed in two passages from the Galenic corpus: one in *Good and Bad Fluids* and another in *Mixtures*. We have demonstrated that although Galen does not explicitly expound on it elsewhere, this distinction is both productive and operative in Galen’s thought, as evidenced in diverse works such as *Anatomical Procedures*, *Natural Capacities* and *The Motion of Muscles*. He emphasizes different concerns in different works, according to the scope and aim of the given treatise; and in many instances, he takes the tripartition of muscles, Natural Moving Organs, and Natural Immobile Organs for granted.72On how different genres elicit different points of view and even what might appear as contradicting claims from Galen, see Singer 1997.

This typology is one of several ways in which Galen distinguishes between parts of the body and classifies them into groups. Others include, for example, the division into natural and psychic organs, or uniform parts as distinct from organs. These in turn can be further divided or labelled, such as the sensory organs, or organs of nutrition as distinct from organs of *pneuma*. These topologies are not contradictory. Parts can receive more than one label, depending on the typology being applied. The different typologies are useful ways for Galen to organize his thoughts and more to the point, to organize his knowledge and explanation of the body, be it for theoretical, clinical or didactic purposes. The typology, that is, the applied model of organization, shifts in accordance to the purpose as well as the topic at hand (e.g. soul, anatomy, physiology, case histories and so forth) and other contexts, such as particular polemics. Some typologies are well-argued and clearly set out by Galen, others he simply applies. Either way, each typology reveals a particular way Galen views and theorizes the body and its parts.

This paper has brought to light another Galenic typology. It appears to be a novel one. Galen adopted some of his typologies from earlier authors, often adapting and elaborating them. The typology presented in this paper is not clearly apparent in other authors, though this merits a deeper investigation. The novelty of Galen’s typology further highlights a question which has loomed throughout this paper. What would motivate or justify such a typology: what purpose does it serve and what is at stake in maintaining it?

Here, two complementary narratives suggest themselves. The first, ‘top-down’ account would prioritize the theoretical stakes at play. Galen cannot consider Natural Moving Organs as muscles because, according to his psychology and physiology, this would render digestion and pulsation voluntary activities.73In *AA*. VII 8 (II 433,5 – 11 Garofalo = II 610 – 611 K.) Galen uses the involuntariness of the pulse as an additional reason, on par with anatomical considerations, to think that the heart is not a muscle. The case of respiration illustrates this point: once he argues that muscles, and not the lungs themselves, enabled respiration, he has to defend the non-evident claim that respiration is a voluntary motion.74See *Mot.Dub*. 2 (128 – 130 Nutton); *Caus.Resp*. 1 – 8 (240 – 244 Furley\Wilkie = IV 465 – 469 K.). Extending similar claims to the activities of the heart and stomach would be nearly impossible to defend and would compromise the tripartition of the soul. What was at stake for Galen according to such a narrative was the tripartition of the soul and its neat and justified mapping upon the body and its workings. But we can also construct a ‘bottom-up’ narrative, wherein Galen observes genuine and systematic anatomical differences between Natural Moving Organs and muscles and between these two groups and Natural Immobile Organs – the very differences we have surveyed above. This distinction demands explanation, especially due to Galen’s teleological view of the body, according to which the anatomical *realia* – shape, structure similarities and differences – must be explained in functional terms. Such an explanation readily emerges from the theoretical framework of the tripartite soul and its physiological implications. In this scenario, instead of the body’s structure explaining or justifying a point about the soul, the focus is reversed and rests on the body itself, its perfectly designed perceptible structure and arrangement and the ultimate functioning it facilitates.

There is no need to choose between these accounts, as they complement rather than contradict each other. What is clear, however, is that once the notion of such a typology – bridging anatomy, physiology, and psychology – was formulated, anatomy played a crucial role in Galen’s justification for it.75We thus gesture towards something like the common distinction between ‘context of discovery’ and ‘context of justification’. See Hoyningen-Huene 1987. We thank an anonymous reviewer for raising this point. Galen emphatically asserts the fundamental role of anatomy in his physiology and psychology, even arguing that meticulous anatomical observations can provide premises (λήμματα or προτάσεις) for demonstrations of physiological and psychological conclusions, following the rigorous standards outlined in Aristotle’s *Posterior Analytics*.76See, e.g., the discussion about the apodeictic value of dissections in *Anatomical Procedures* (*AA*. II 2 [I 77,9 – 13 Garofalo = II 286 K.]) and the discussions in Barnes 1991; Hankinson 1994; Lloyd 1996; 2005; Rocca 2003, 49 – 58. While this attempted marriage between anatomy and deductive demonstration has its problems, Galen’s position illuminates his commitment to grounding physiological and psychological theories in anatomical claims. Although anatomical observation is never pre-theoretical and theoretical demands have their effects, we must acknowledge the role that precise anatomical details play in defending his views, regardless of their genesis.77Galen acknowledges the significance of reasoning in deriving accurate conclusions from anatomical phenomena, which provide an indication (ἔνδειξις) that should be further elaborated. See Rocca 2003, n. 35 for discussion and references.

Indeed, at least in the typology of organs we have surveyed, Galen appears to ground his physiological and psychological arguments in a set of anatomical differences he observes in the body. Attention to the interplay and complementarity of anatomy, physiology, and psychology on the one hand, and the fundamental role anatomy plays in justifying claims in other domains on the other, sheds new light on Galen’s unique mode of argumentation as a philosophically informed physician.78On Galen’s complicated relationship with philosophy and philosophers see Singer 2014. Singer’s analysis only bolsters the claim that even when reading Galen in his most theoretical mode, we should never forget his commitment to be a philosophical physician, i.e., one that distinguishes himself from his day’s professional philosophers exactly by his reliance on anatomical and otherwise observable premises about the body.
